# Parallax-Robust Surveillance Video Stitching

**DOI:** 10.3390/s16010007

**Published:** 2015-12-25

**Authors:** Botao He, Shaohua Yu

**Affiliations:** 1 School of Optical and Electronic Information, Huazhong University of Science and Technology, Wuhan 430074, China; 2 Wuhan Research Institute of Posts and Telecommunications, Wuhan 430074, China; shyu@fhzz.com.cn

**Keywords:** video stitching, video surveillance, layered warping, parallax

## Abstract

This paper presents a parallax-robust video stitching technique for timely synchronized surveillance video. An efficient two-stage video stitching procedure is proposed in this paper to build wide Field-of-View (FOV) videos for surveillance applications. In the stitching model calculation stage, we develop a layered warping algorithm to align the background scenes, which is location-dependent and turned out to be more robust to parallax than the traditional global projective warping methods. On the selective seam updating stage, we propose a change-detection based optimal seam selection approach to avert ghosting and artifacts caused by moving foregrounds. Experimental results demonstrate that our procedure can efficiently stitch multi-view videos into a wide FOV video output without ghosting and noticeable seams.

## 1. Introduction

**Figure 1:**
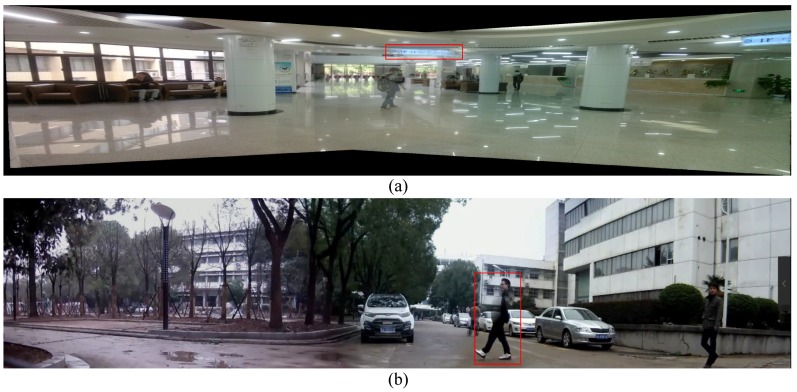
The main reason for ghosting comes from misalignment error. Here we show two types of ghosting in red boxes: (**a**) ghosting caused by using a global projective warping; (**b**) ghosting caused by persons moving across seams.

Image stitching, also called image mosaicing or panorama stitching, has received a great deal of attention in computer vision [1,2,3,4,5,6,7,8]. After decades of development, the fundamentals of image stitching are well studied and relatively mature now. There are many research works on image stitching [1,2,3,4,5,6,7,8], and it is typically solved by estimating a global 2D projective warp to align the input images. A 2D projective warp uses a homography parameterized by 3×3 matrices [1,2,3,9], which can preserve global image structures, but cannot handle parallax. It is correct only if the scene is planar or if the views differ purely by rotation. However, in practice, such conditions are usually hard to satisfy, thus ghosting and seams yield (see Figure 1). If there is parallax in input images, no global homograhpy exists that can be used to align these images. When a global warp is used to stitch these images, ghosting like Figure 1a would appear. Some advanced image composition techniques such as seam cutting [10,11,12] can be used to relieve these artifacts. However, if there are moving objects across the seams, another kind of ghosting like Figure 1b would yield.

Previous research indicates that one of the most challenging problems to create seamless and drift-free panoramas is performing a correct image alignment rather than using a simple global projective model and then fix the alignment error [6,8,9]. Thus, some recent image stitching methods focus on using spatially-varying warping algorithms to align the images [6,7,8], these methods can handle parallax and allow for local deviation to some extent but require more computation.

With wide applications in robotics, industrial inspection, surveillance and navigation, video stitching faces all the problems as image stitching does and can be more challenging due to moving objects in videos. Some researchers tried to build panoramic images by aligning video sequences [13,14], which is panoramic image generation rather than expansion of the FOV of dynamic videos. Other works focus on freely moving devices [15,16,17,18], especially mobile devices, which include techniques such as efficient computation of temporal varying homography [15], optimal seam selection for blending [16], and so on. However, due to complex computation and low resolution, they may not be suitable for surveillance application.

**Figure 2:**
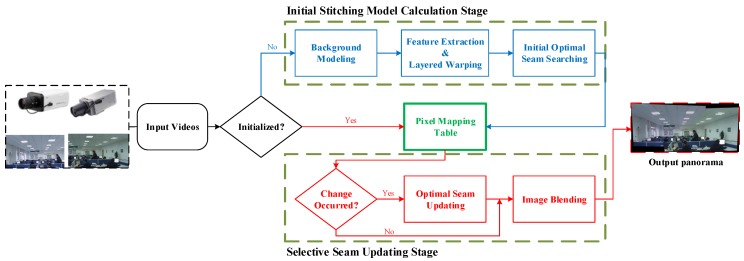
Outline of the proposed video stitching procedure. Our method consists of two stages: initial stitching model calculation stage and selective seam updating stage. In the initial stitching model calculation stage, we first use background modeling algorithm to generate still background of each input source video, then utilize layered warping algorithm to align background images, finally, we perform optimal seam searching and image blending to generate panorama backgrounds. The resulting stitching model is a mapping table in which each entry indicates the correspondence between the pixel index of source images and that of panorama image. To relieve the ghosting effect caused by moving objects, at selective seam updating stage, we perform seam updating according to whether there are objects moving across previous seams or not. Our method reaches a balance between suppressing ghosting artifacts and real-time requirement.

In this paper, we present an efficient parallax-robust surveillance video stitching procedure that combines layered warping and the change-detection based seam updating approach. As the alignment validity of video stitching is still crucial as it is in seamless image stitching, we use a layered warping method for video registration instead of a simple global projective warp. Xiao *et al*. [19] provides a similar layer-based video registration algorithm, but it aims at aligning a single mission video sequence to the reference image via layer mosaics and region expansion, rather than building a dynamic panoramic video for motion monitoring. Through dividing matched feature pairs into multiple layers (or planes) and local alignment based on these layers, the layered warping method seems to be more robust to parallax. Moreover, the warping data for fixed surveillance videos are stored in an index table for “recycling-use” in subsequent frames to avoid repeated registration and interpolations. This index table is referred to as the initial stitching model in this paper. Aside from layered warping, a local change-detection based seam updating method for overlapping regions is performed to disambiguate the ghosting caused by moving foregrounds. Figure 2 shows the video stitching process presented in this paper.

## 2. Related Works

Recently, video stitching has drawn a lot of attentions [11,20,21] due to its wide usage in public security. Generally speaking, surveillance video stitching can be regarded as image stitching for every individual frame since the camera positions are always fixed. Different from image stitching technologies, video stitching requires more strict real-time processing ability, and large parallax and dynamic foregrounds must be carefully considered to obtain consistent wide field of view videos. Image stitching is relatively a well studied problem in computer vision [1,9,22]. Several freewares and commercial softwares are also available for performing image stitching, like AutoStitch [23], Microsoft’s Image Compositing Editor [24], and Adobe’s Photoshop CS5 [25] mosaicing feature. However, these approaches all work under the assumption that the input images contains little or no parallax, which implies that the scene is either sufficiently far away from the camera to be considered planar, or that the images have been taken from a camera carefully rotated about its center of projection. This assumption is too strict to be satisfied in real surveillance scenarios. Thus, misalignment artifacts like ghosting or broken image structures will make the final panorama visually unacceptable (see Figure 1).

Some advanced image composition techniques, such as seam cutting [10,11,12] and blending [26,27], have been employed to reduce the artifacts. However, these methods alone still cannot handle significant parallax. In this paper, we also use seam cutting and blending as the final steps to suppress artifacts. Recent studies on spatially-varying warping are another way out [6,7,28]. As-projective-as-possible (APAP) warps [7] employed local projective warps within the overlapping regions and performed moving direct linear transformation [29] to smoothly extrapolate local projective warps into the non-overlapping regions. Shape-Preserving Half-Projective (SPHP) warp [28] spatially combines a projective transformation and a similarity transformation and has the strengths of both. However, instead of improving alignment accuracy, its main concern is to decrease distortion of non-overlapping area caused by the projective transformation. So even if it introduces APAP [7] into their warp, they cannot solve the problem of structure distortion in the overlapping area in APAP [7]. Gao *et al.* [6] proposed to uses a dual homography warp (DHW) algorithm for scenes containing two dominant planes (ground plane and distant plane). While it performs well if the required setting is true, it may fail when there are more than two planes in the source images. Inspired by DHW [6], we propose to use a layered warping algorithm to align the background scenes, which is location-dependent and turned out to be more robust to parallax than the traditional global projective warping methods and more flexible than DHW [6] which can only process images with two planes.

Apart from parallax, moving foregrounds are another reason for ghosting in video stitching. Although we propose to use layered warping to align images as accurate as possible and to utilize seam cutting to composite source images, some artifacts may still exist when objects move across the seam. Liu *et al*. [20] only used the stitching model calculated with first few frames to stitch following frames and didn’t consider the moving foregrounds. In contrast, Tennoe *et al*. [30] and Hu *et al*. [11] update the seam in every frame, which is very time-consuming. To balance between suppressing the artifacts and the real-time requirement, we propose to first detect changes around the previous seams, and only perform seam update when there are moving objects across seams. Since the price of change detection around seams is much lower than that of updating seams, artifacts caused by moving foregrounds can be suppressed with acceptable time consumption by our method.

## 3. Initial Stitching Model Calculation

Since our focus is on improving image alignment accuracy and reducing artifacts caused by moving objects, we do not change the conventional pipeline [9] of image stitching with different number of input sources. For ease of illustration, in the following text, we only describe the layered warping algorithm and selective seam updating algorithm with two input videos. In the experiment section, we provide stitching results with both two and more than two input videos.

### 3.1. Background Image Generation and Feature Extraction

For fixed surveillance cameras, we only perform alignment at the stitching model calculation stage because the computation of temporal varying homography may result in palpable jitter of background scenes in the panoramic video. Background modeling is essential to avoid volatile foreground [31,32]. We take the first $N_{gmm}$ frames of input videos to establish the background frame utilizing the Gaussian Mixture Model (GMM) [33]. SIFT [3,34] features of the background frame is extracted and matched into pairs through Best-Bin-First (BBF) algorithm [35].

### 3.2. Layered Warping

Inspired by DHW [6], we assume that different objects in a scene usually lie in different depth layers, the objects in the same layer (plane) shall be consistent with each other in spatial transformation. Compared to warping using a global homography or warping using dual homography, layered warping may be more adequate and robust for abundant scenes.

Layer registration. We denote the input images as $I_{1}$ and $I_{2}$ respectively, and the matching feature pairs as $F={\left\{\left(p_{i}^{1},p_{i}^{2}\right)\right\}}_{i=1}^{N}$, where $p_{i}^{k}$ is the coordinate of the *i*-th matching point from $I_{k}$ (k=1,2). Given the matching feature pairs, we first utilize Random Sample Consensus (RANSAC) algorithm [36] to robustly group the feature pairs into different layers, then estimate the homography for each layer. Denote the consistent matching feature pairs of layer *k* as $L_{k}$, the number of matches in $L_{k}$ as $|L_{k}|$ and its corresponding homography as $H_{k}$. To guarantee the grouped layer to be representative, we introduce a threshold $N_{min}$ which denotes the minimum number of matching pairs in $|L_{k}|$. Layers whose number of matching pairs is smaller than $N_{min}$ are simply dropped. The detailed layer registration process is presented in Algorithm 1.

**Algorithm 1** Layer registration utilizing multiple-layer RANSAC**Input:** Initial pair set $F_{1}=F$, threshold $N_{\min}$ and iteration index k=0;**Output:** Each layer’s matching pair set $L_{k}$ and its corresponding homography $H_{k}$; **repeat**  k←k+1  RANSAC in pair set $F_{k}$ for model $p_{k_{n}}^{1}\times Hp_{k_{n}}^{2}=0$, where $\left(p_{k_{n}}^{1},p_{k_{n}}^{2}\right)\in F_{k}$;  Divide outliers $V_{out}$ and inliers $V_{in}$ according to H;  **if**
$|V_{in}|\geq N_{min}$
**then**   Set matching pair set of the *k*-th layer as $L_{k}=V_{in}$;   Set homography of the *k*-th layer as $H_{k}=H$;  **end**
**if**  Set the pair set of next iteration as $F_{k+1}=V_{out}$; **until**
$|F_{k+1}|<N_{\min}$

**Figure 3:**
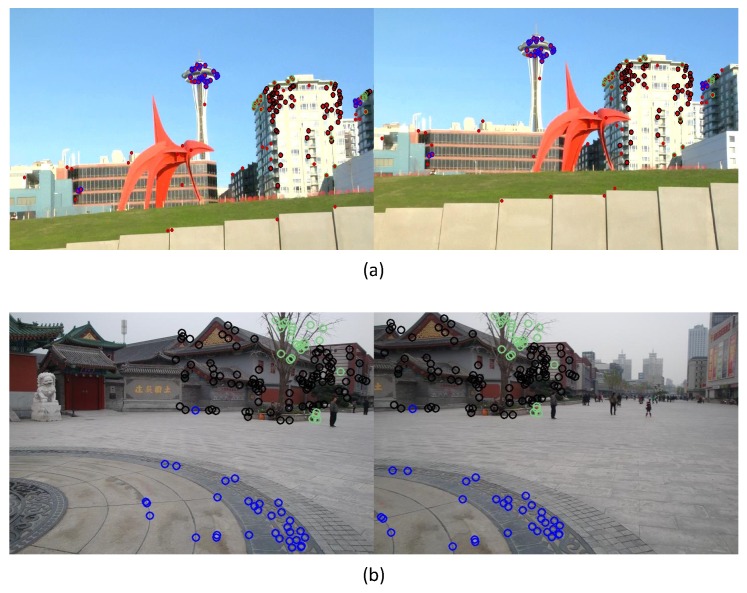
Examples of matched feature points in multiple layers, different color indicate different layers: (**a**) multiple-layer matched feature pairs of images with large parallax; (**b**) multiple-layer matched feature pairs of images with a distant plane and ground plane

Through the layer registration process, we divide the matched feature pairs to multiple layers, each of which contains a set of feature pairs that are consistent with a common homography. Figure 3 shows two examples of layer registration, in which feature points are illustrated as circles and points with the same color are from the same layer. From Figure 3 we can see that the grouped matching pairs of the same layer are almost from the same plane or with the same depth, which is in accordance with our expectation.

Local alignment. To simplify calculations of local alignment, the source image is divided into M×N grids. Since a feature point is not usually coincident with any grid vertex, we use the distances between the grid center and its nearest neighbors in different layers to vote on the warp of the due grid. A grid $g_{j}$ is represented by its center point $c_{j}$. The homography of the grid $g_{j}$, denoted by $H_{j}^{*}$, is computed by fusing $H_{k}$ of multiple layers using a weight $w_{k}^{j}$ by Equation (1)

(1) $$H_{j}^{*}=\sum_{k}w_{k}^{j}H_{k}$$

where $w_{k}^{j}=a_{k}^{j}/\sum_{i}a_{i}^{j}$ and $a_{k}^{j}$ is a position dependent Gaussian weight:

(2) $$a_{k}^{j}=\exp\left(-\frac{\left|\right|c_{j}-p_{k}^{*}{\left|\right|}^{2}}{\sigma^{2}}\right)$$

Here $p_{k}^{*}$ denotes the nearest neighboring feature point of $c_{j}$ in layer *k* and *σ* is a scale constant.

After deriving the local homography for each grid, the target pixel position $p^{\prime }$ in the reference image for the source pixel at position p in grid $g_{j}$ can be easily obtained by Equation (3):

(3) $$p^{\prime }=H_{j}^{*}p$$

**Figure 4:**
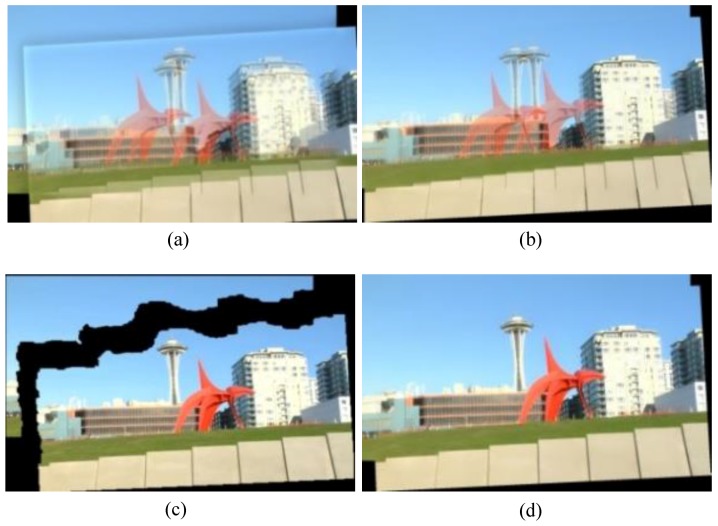
Examples of panoramas with (**a**) global projective warp and (**b**) layered warp, and seam selection results in (**c**). (**d**) is the final fused image according to selected seam in (**c**). The seam selection result (**c**) is based on stitched image (**b**) with layered warp.

This process is referred to as forward mapping [9]. To accelerate the computation, we only perform forward mapping once, and store the correspondence between source pixel positions and target pixel positions in the pixel mapping table. This pixel mapping table is exactly the stitching model. The index tables are stored so that the warped image can be obtained by looking up each corresponding pixel in the source image instead of repeated transformation when new frames arrive. Figure 4a,b shows the panorama images with global projective warp and layered warp respectively. It is clear that the building in Figure 4b is better aligned than that in Figure 4a.

### 3.3. Optimal Seam Cutting

Though layered warping is parallax-robust, it is applied only in the stitching model calculation stage on the extracted background scene. We perform the seam selection method to disambiguate the ghosting caused by the moving foreground.

Optimal seam searching method, also called optimal seam selection [5] or seam cutting, is to search for an optimal seam path which is a pixel-formed continuous curve in the overlapping area to connect pairwise warped images.

The seam should neither introduce inconsistent scene elements nor intensity differences. Therefore, two criteria are applied in this paper to form the difference map of overlaps: the intensity energy $E_{ij}^{C}$ and gradient energy $E_{ij}^{g}$ which are defined as:

(4) $$\left\{\begin{array}{cc} E_{ij}^{C} & =\frac{\left|\right|I_{A}\left(i,j\right)-I_{B}\left(i,j\right)\left|\right|}{\max(I_{A}\left(i,j\right),I_{B}\left(i,j\right))}\\ E_{ij}^{g} & =||\nabla I_{A}\left(i,j\right)-\nabla I_{B}\left(i,j\right){\left|\right|}^{2} \end{array}\right.$$

Here $I_{A}\left(i,j\right)$, $I_{B}\left(i,j\right)$, $\nabla I_{A}\left(i,j\right)$ and $\nabla I_{B}\left(i,j\right)$ are the intensity and gradient of pixel (i,j) in image A and B respectively. Finding the optimal stitching seam is an energy minimization problem (see Equation (5)) and can be converted to a binary Markov Random Field (MRF) labeling problem [37]:

(5) $$\arg\min_{ij}\sum_{ij}\left(E_{ij}^{C}+\lambda E_{ij}^{g}\right)$$

To accelerate the computing process, the warped images and background images are down sampled before seam selection and restored to the original size after seam selection in our procedure. Figure 4c and d show an example of seam selection. From Figure 4c we can see, the selected seam mainly crosses flat areas with little gradients or intensity differences, thus the resulted panorama is visually consistent with no noticeable ghosting.

## 4. Selective Seam Updating

After initial stitching model calculation, the videos are overall pre-aligned. However, for moving foregrounds, the previous seam may lose its optimality or even miss information. Since the seam cutting algorithm requires complex computation even on the down-sampled frames, it is difficult to be used to update video frames in real-time. So we perform a change detection based seam updating method instead of real-time seam selection.

### 4.1. Change Detection around Previous Seams

**Figure 5:**
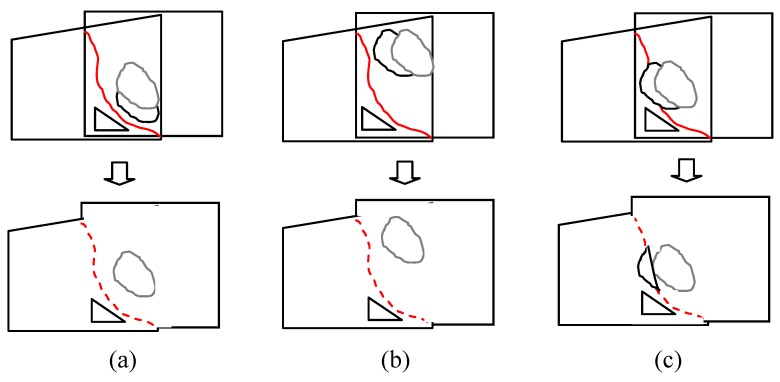
(**a**) The original aligned frame with a stitching line; (**b**) newly aligned frame with changes not cross the line; (**c**) frame with changes cross the line.

First of all, we resize the new warped frames to a smaller scale as we did in the optimal seam selection section. Even if the images have been scaled down, direct calculation of the gradient of the two images and evaluation of the change may be expensive. However, we observed that compared with changes in non-overlapping area, those in the overlapping area are more likely to violate the optimal seam. Furthermore, only changes across the optimal seam may result in the failure of it (see Figure 5), which cam be measured by gradient difference.

As an optimal seam is searched, the original gradient value set ${\tilde{G}}_{0}$ of the seam is stored. We set ${\tilde{g}}_{i0}$ as the original gradient of pixel $p_{i}$ in the present seam, and ${\tilde{g}}_{it}$ as the gradient in time *t*. To calculate the number of pixels that have large gradient variations, we use the following rule to judge if changes occured at pixel $p_{i}$ or not:

(6) $$C_{t}=\left\{p_{i}|\frac{{\tilde{g}}_{it}-{\tilde{g}}_{i0}}{{\tilde{g}}_{i0}}>\delta \right\}$$

If the total pixel number in $C_{t}$ is bigger than $N_{cd}$, we consider that there are new moving objects in the overlapping area and the optimal seam shall be updated. In Equation (6), *δ* is a constant chosen empirically, whereas $N_{cd}$ depends on $\tilde{N}$, which is the total pixel number of the seam. We set it as $0.3\tilde{N}$ in our experiments.

### 4.2. Seam Updating

For each new warped frame, change detection, as described in the previous section, is performed to see if an alteration of the seam is needed, if so, we select a new seam by the seam selection algorithm presented in Section 3.3, otherwise, we continue to use the previous seam and move on to the image blending process.

### 4.3. Blending

Seam cutting can eliminate ghosting, but it provides images without overlaps which may result in noticeable seams. We expand the seamed image with a spherical dilating kernel, and use a simple weighted linear blending method [9] to blend the images.

## 5. Experiments

To evaluate the performance of our video stitching procedure, we conduct some experiments on both still images with parallax and actual surveillance videos.

### 5.1. Experimental Settings

There are several empirical parameters which should be manually tuned for different cases. At the background modeling stage, we use the initial 20 frames to construct a GMM model with 5 components for each pixel. The frame number may be set larger if the scenes are more cluttered. In our experiment, $N_{min}$ is set as 12, which is the minimum number of matching pairs for each layer, and the source 720P image is divided into 80×45 grids for layered warping. We provide stitching results with both two, three and four channels as input.

### 5.2. Stitching Still Images

**Figure 6:**
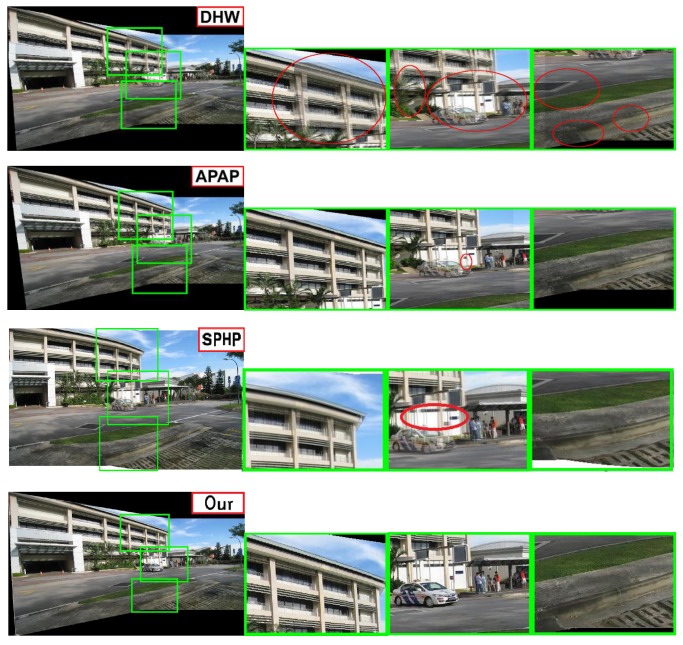
Comparisons among dual homography warp (DHW) [6], as-projective-as-possible (APAP) [18], Shape-Preserving Half-Projective (SPHP) [28] and Our algorithm, Red circles highlight errors.

**Figure 7:**
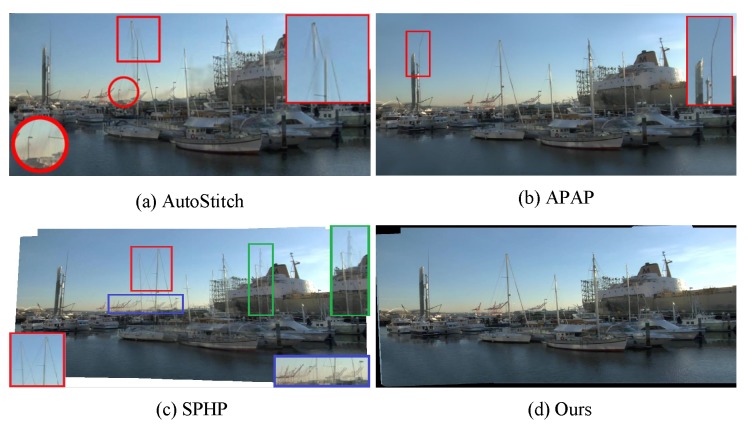
Comparisons among various stitching methods. colored rectangles and their zoom-in rectangles illustrate the false misalignment.

To evaluate the proposed layered warp algorithm, we first conduct experiments on still images with parallax and compare our results with other image stitching methods in Figure 6 and Figure 7.

DHW [6] only considers two layers (a distant plane and a ground plane), so it fails when the scene has multiple depth layers or contains complex objects. Our algorithm is more adequate and robust than DHW for abundant scenes (see Figure 6). Figure 7 shows the comparison among our algorithm and some state-of-art algorithms on a tough scene from [8]. As APAP tries to align two images over the whole overlapping region, it distorts the salient image structure, such as the pillar indicated by the red rectangle. SPHP also fails when the overlapping area covers most of the image and there is large parallax in it (see Figure 7c). From Figure 6 and Figure 7 we can see, the stitching results of our algorithm contain no noticeable distortions and are more visually acceptable than other methods.

### 5.3. Stitching Fixed Surveillance Videos

**Figure 8:**
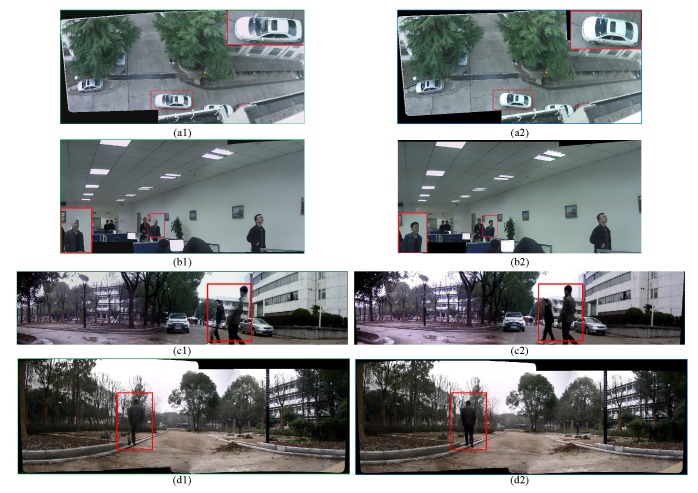
Frames taken from the output Field-of-View (FOV) videos: (**a1**–**d1**) are taken from the output videos using initialized seam without updating. (**a2**–**d2**) are taken from the output video using change-detection based seam updating approach. (**a1**), (**a2**), (**b1**) and (**b2**) are the stitching results of two channels of 720P videos. (**c1**) and (**c2**) are the stitching results of three channels of 720P videos. While for (**d1**) and (**d2**), they are the stitching results of four 720P videos.

To evaluate the effectiveness of selective seam updating strategy, we perform extensive experiments on fixed surveillance videos, and compare our results with that of without seam updating. The results are shown in Figure 8, from which we can see, when we do not perform seam updating in the video stitching stage, some obvious artifacts caused by moving objects would make the stitched video visually unacceptable. The comparison results indicate that the change detection based seam updating approach is rather helpful to avert ghosting and mis-alignments caused by moving targets, especially when there are moving objects across previous seams.

### 5.4. Time Analysis

Apart from surpressing ghosting caused by parallax and moving targets, the real-time requirement of video stitching task should also be seriously considered when developing algorithms. To evaluate the speed of the proposed method, we take two videos as input and test the time consumption for different video resolutions. To make a fair comparison, the speed of stitching with temporal varying homography, layered warping without selective seam updating and layered warping with selective seam updating are all listed in Table 1. These experiments are all conducted on a PC with Intel Core i3 2.33 GHz CPU with two cores and 2GB RAM. All tested algorithms are implemented with C++.

From Table 1 we can see, as the index table has already been established according to the registration result in the stitching model calculation stage, frames can be projected to the panoramic frame by directly indexing instead of alignment and mapping for every new frame, thus the stitching efficiency of the proposed method is greatly improved. Table 1 also shows that the selective seam updating process slows down the frame stitching process to some extent.

**Table 1:** Comparisons of the speed among different video stitching methods on input-videos with different resolutions.

Resolution	Stitching with Temporal Varying Homography	Proposed Algorithm without Seam Updating	Proposed Algorithm with Seam Updating
720p: 1280 × 720	4.764 s	0.051 s	0.083 s
480P: 720 × 480	2.326 s	0.035 s	0.045 s
CIF: 352 × 288	1.025 s	0.021 s	0.032 s

## 6. Conclusions

This paper presents an efficient parallax-robust stitching algorithm for fixed surveillance videos. The proposed method consists of two parts: alignment work done at the stitching model calculation stage and change detection based frame updating at the video stitching stage. The algorithm uses layered warping method to pre-align the background scene which is robust to scene parallax. As for each new frame, an efficient change detection based seam updating method is adopted to avert ghosting and artifacts caused by moving foregrounds. Thus, the algorithm can provide good stitching performance with no ghosting and artifacts for dynamic scenes efficiently.
